# Influence of Harvesting Method on Essential Oil Composition, Antioxidant Activity, and Operational Efficiency in Anatolian Sage (*Salvia fruticosa* Mill.)

**DOI:** 10.3390/molecules31122023

**Published:** 2026-06-09

**Authors:** Sadiye Ayşe Çelik

**Affiliations:** Faculty of Agriculture, Department of Field Crops, Division of Medicinal Plants, Selçuk University, Konya 42130, Turkey; sacelik@selcuk.edu.tr; Tel.: +90-5397993363

**Keywords:** *Salvia fruticosa*, Anatolian sage, harvesting method, essential oil composition, phenolic compounds, antioxidant activity

## Abstract

*Salvia fruticosa* Mill. (Anatolian sage) is an important medicinal and aromatic plant widely valued for its essential oil and phenolic compounds. Harvesting practices may influence both biomass yield and the chemical quality of plant raw materials. This study evaluated the effects of manual and machine harvesting on selected physical characteristics, essential oil composition, mineral content, and antioxidant-related phytochemical properties of *S. fruticosa* cultivated under Central Anatolian conditions, together with the operational performance of both harvesting methods. Manual harvesting resulted in higher fresh and dry biomass yields and a greater essential oil content (2.03%) compared with machine harvesting (1.57%). Mineral analysis showed that Ca, Zn, Cu, and B concentrations were higher in manually harvested samples, whereas K and Mg contents were slightly higher in machine-harvested plants. Essential oil characterization demonstrated that 1,8-cineole was the dominant compound and its proportion differed markedly between harvesting methods, reaching 43.07% in manual harvesting and 21.77% in machine harvesting. Antioxidant activity determined by the DPPH assay was 0.093 mg TE mL^−1^ for manual harvesting and 0.096 mg TE mL^−1^ for machine harvesting. Additional phytochemical analyses revealed total phenolic contents of 134.6 and 129.3 mg GAE g^−1^ extract, total flavonoid contents of 22.7 and 25.2 mg QE g^−1^ extract and FRAP values of 382 and 336 µmol Fe^2+^ g^−1^ extract for manual and machine harvesting, respectively. These findings indicate that harvesting technique affects certain compositional parameters but causes only limited changes in the overall antioxidant potential of *S. fruticosa* extracts.

## 1. Introduction

Medicinal and aromatic plants (MAPs) constitute strategic botanical resources for the global pharmaceutical, cosmetic, food, and functional product industries owing to their rich reservoir of secondary metabolites and bioactive constituents. The economic value of these crops is closely associated with specific quality parameters, particularly essential oil (EO) yield, chemical composition, and antioxidant capacity. Within the Lamiaceae family, which is widely recognized for its high essential oil productivity, Anatolian sage (*Salvia fruticosa* Mill.; syn. *S. triloba* L.) represents a species of considerable ecological adaptability and commercial importance. *S. fruticosa* is known by several vernacular names throughout its distribution area. In Türkiye, it is commonly referred to as “Anadolu adaçayı”, whereas in the international herbal trade it is frequently marketed as “Greek sage” or “East Mediterranean sage.” The essential oil obtained from this species is traditionally known in Türkiye as “elma yağı” or “acı elma yağı,” reflecting its characteristic aroma and widespread ethnomedicinal use [1].

Traditionally, Anatolian sage essential oil has been used as a carminative, antispasmodic, mild sedative, and digestive aid, particularly for the relief of gastrointestinal discomfort and infantile colic. These therapeutic properties are largely attributed to its high content of oxygenated monoterpenes, especially 1,8-cineole and camphor, which are known to exhibit antimicrobial, anti-inflammatory, and smooth muscle–relaxant activities [2,3]. Although not endemic to Türkiye, *S. fruticosa* is a characteristic component of the Eastern Mediterranean flora and is naturally distributed across Türkiye, Greece, Cyprus, Italy, Syria, Israel, and parts of North Africa. While Türkiye represents a major center of diversity for the genus *Salvia* L., hosting numerous endemic taxa, *S. fruticosa* is distinguished by its economic importance rather than endemicity. It remains one of the most commercially exploited sage species in the region and constitutes a major biological source for large-scale essential oil production [1,2,3,4].

Morphologically, *S. fruticosa* is a perennial evergreen subshrub reaching 60–120 cm in height, characterized by quadrangular stems and gray-green rugose leaves densely tomentose on the abaxial surface. The epithet “triloba” refers to its frequently trilobed leaves consisting of a prominent terminal lobe and two smaller basal lobes. The plant produces verticillaster inflorescences bearing bilabiate flowers ranging from lilac to violet blue. In Türkiye, the species occurs predominantly on calcareous slopes and lithosols within Aegean and Mediterranean maquis ecosystems, extending from sea level to elevations of approximately 1000 m [4].

The essential oil of *S. fruticosa* is characterized by a predominance of oxygenated monoterpenes, particularly 1,8-cineole, camphor, and borneol, which largely determine its pharmacological and aromatic value [5]. However, the biosynthesis and accumulation of these metabolites are highly dynamic and depend on complex interactions between genetic background, environmental conditions, and agronomic practices. Previous studies have demonstrated that environmental stress factors, harvest timing, and post-harvest handling can significantly influence both 1,8-cineole concentration and total EO yield [3,4,6].

In MAP cultivation systems, harvesting represents a critical operational stage that directly determines biomass recovery, drug yield, and phytochemical integrity [7,8]. The glandular trichomes responsible for EO synthesis and storage are particularly sensitive to mechanical damage. Consequently, traditional production systems have relied predominantly on manual harvesting to minimize tissue disruption and preserve volatile compounds. However, increasing global demand, rising labor costs, and shortening harvest windows have encouraged the adoption of mechanized harvesting systems [9]. Mechanical harvesting enables rapid biomass collection across large production areas; nevertheless, it may introduce physical stresses such as vibration, impact, friction, and non-selective cutting, which can potentially affect plant tissues and volatile compound retention prior to distillation [10]. In *S. fruticosa*, its major constituent 1,8-cineole has been reported to be particularly sensitive to harvesting and processing conditions [3].

Beyond essential oil parameters, harvesting strategy may also influence biomass distribution, plant fraction ratios, and physiological responses. Mechanical stress can modify plant metabolic responses and may influence the accumulation of secondary metabolites, including phenolic compounds. Phenolics and flavonoids represent major classes of secondary metabolites in *Salvia* species and are widely recognized for their contribution to antioxidant activity and biological properties. Therefore, the determination of total phenolic content (TPC), total flavonoid content (TFC), and ferric-reducing antioxidant power (FRAP) provides valuable insight into the antioxidant-related phytochemical profile of plant extracts and allows the evaluation of potential variations associated with different harvesting techniques. Antioxidant capacity of plant extracts is commonly assessed through methods such as DPPH radical scavenging activity and FRAP assays, which reflect the reducing potential associated with phenolic constituents [11,12].

Although Türkiye is one of the principal natural distribution centers of *Salvia fruticosa*, commercial-scale field cultivation of this species has not yet been established in the country. The raw material entering both domestic and international herbal trade is predominantly obtained from wild populations, and the few cultivation attempts have failed to expand to a commercial level because harvesting is performed almost entirely by hand. The intensive labor requirement, the narrow harvest window, and the increasing difficulty of securing seasonal agricultural workers constitute the main bottlenecks preventing large-scale production. Consequently, the expansion of *S. fruticosa* cultivation to wider production areas is closely linked to the resolution of mechanization problems at the harvesting stage, which currently represents the principal constraint to commercial sage production in Türkiye. In this context, evaluating the impact of mechanical harvesting on yield and phytochemical quality becomes a prerequisite for the sustainable commercial cultivation of the species and provides the central rationale of the present study.

In economic terms, sage (*S. fruticosa* together with the accompanying species *S. tomentosa*) represents one of the most important medicinal and aromatic plant export commodities of Türkiye. According to Turkish Statistical Institute (TÜİK) records, Türkiye exported approximately 2176 tons of dried sage leaves with a foreign-currency earning of about 8.16 million USD in 2020, while the five-year (2016–2020) average reached around 2100 tons per year; in addition, roughly 27.4 tons of sage essential oil were exported in 2020, generating about 0.17 million USD of revenue [13,14]. Between 2010 and 2020, Turkish sage was marketed to more than 60 countries, with the United States, Germany, France, Italy, and Spain being the principal destinations [13]. Despite this strong export performance, nearly the entire production chain still relies on wild collection from natural populations—especially in the Mediterranean and Aegean provinces such as Hatay, Mersin, Antalya, and Muğla—because commercial-scale field cultivation has not been established and no officially registered cultivar exists in the country [13]. This heavy dependence on wild harvesting, together with increasing international demand, highlights the strategic importance of developing mechanizable cultivation systems to secure the sustainable supply and export potential of Anatolian sage.

Despite Türkiye’s prominent position in the global sage market, comparative studies evaluating manual versus mechanized harvesting in *S. fruticosa*, particularly under the semi-arid transitional conditions of Central Anatolia, remain limited. The present study was conducted in the Göztepe region of Karaman (alt. ~1050 m), characterized by high solar irradiance, marked diurnal temperature variation, and low relative humidity—conditions conducive to secondary metabolite accumulation. Focusing on *S. fruticosa* (Anatolian sage), this study evaluates the effects of manual and mechanical harvesting on pre- and post-harvest physical properties, mineral composition, essential oil yield and composition, and antioxidant-related phytochemical parameters, including DPPH radical scavenging activity, total phenolic content (TPC), total flavonoid content (TFC), and FRAP antioxidant capacity. In addition, the operational performance of both harvesting methods was determined in order to provide a scientific basis for sustainable and quality-oriented harvesting strategies in medicinal plant production.

Beyond confirming general trends already reported for other Mediterranean sage populations, the specific added value of the present work lies in three complementary aspects that, to the best of our knowledge, have not previously been combined in a single study on *S. fruticosa*. First, the harvesting-method comparison is examined through the joint application of univariate inference (Welch-corrected pair-wise tests) and multivariate ordination (PCA), which moves the analysis beyond the single-parameter comparisons that still dominate the essential-oil chemodiversity literature and allows method-induced compositional shifts to be assessed as a coherent signature rather than as isolated mean differences. Second, the dataset assembled here is intentionally comprehensive: agronomic descriptors (biomass yield, plant fractions, harvest losses), chemical descriptors (essential oil content and full GC–MS profile, mineral composition, TPC, TFC, DPPH, FRAP) and operational descriptors (effective field capacity, fuel consumption, labor requirement) of both harvesting methods were obtained on the same plant material in the same season, providing an integrated agronomic–chemical–operational evaluation that is rarely available for medicinal Lamiaceae. Third, the trial was carried out under the semi-arid transitional climate of Central Anatolia (Karaman, ~1050 m a.s.l.), an agro-ecological zone that differs markedly from the coastal Mediterranean environments where most of the existing *S. fruticosa* harvesting and chemodiversity data have been generated, and which represents the most realistic context for the prospective expansion of large-scale, mechanizable sage cultivation in Türkiye. Together, these three elements define the specific contribution of the present study and frame the hypotheses tested below.

On this basis, we formulated three testable hypotheses, moving beyond the descriptive univariate comparisons that still dominate the essential-oil chemodiversity literature [15,16]: (i) mechanical harvesting of *S. fruticosa* produces a coherent, multivariate shift in the raw-material composition that cannot be captured by single-parameter comparisons; (ii) the mechanical cutting process, being non-selective, incorporates a larger proportion of lignified stems and older tissues into the harvested biomass, which are known to be depleted in leaf-sink, phloem-immobile elements such as calcium, zinc and copper, and the resulting dilution should be detectable as a coherent reduction in these elements in the machine-harvested material; and (iii) once the multivariate separation between the two harvesting methods has been established, the antioxidant endpoints (DPPH, FRAP, TPC, TFC)—frequently cited as quality drivers—contribute only marginally to the overall chemical signature. To the best of our knowledge, this is the first study that integrates full triplicate raw data, pair-wise Welch-corrected tests for every parameter, and principal component analysis (PCA) to disentangle method-induced compositional changes in Anatolian sage.

## 2. Results and Discussion

The results of the analyses conducted to evaluate the effects of harvesting methods on the morphological, yield, and quality parameters of *S. fruticosa* are discussed sequentially.

### 2.1. Some Pre- and Post-Harvest Physical Characteristics

The mean plant height, canopy diameter, and tillering count of the harvested plants were determined as 638.3 mm, 823.3 mm, and 53 branches, respectively. The mean fresh and dry herbage weights per plant averaged 600 g plant^−1^ and 220 g plant^−1^, respectively. In manual harvesting, the average fresh and dry herbage yields averaged 6670 kg ha^−1^ and 2440 kg ha^−1^, respectively, whereas in mechanical harvesting, these values were found to be 5800 kg ha^−1^ and 2120 kg ha^−1^. Post-harvest measurements revealed that the average stubble height was 223.3 mm for mechanical harvesting and 160 mm for manual harvesting. Regarding the post-harvest plant canopy diameter, the mean value was measured as 488.3 mm in mechanical harvesting, whereas it was 588.3 mm in manual harvesting. Since manual harvesting involved a lower cutting height (cutting closer to the ground) compared to mechanical harvesting, the biomass yield per unit area significantly increased. An evaluation of the harvest loss results revealed that the total loss rate in mechanical harvesting (12.04%) was approximately 50% higher than that of manual harvesting (8.40%). This significant increase is attributed to specific structural and operational parameters, including vibration generated during harvesting, machine forward speed, impact forces, and the inability of the fixed cutting height to fully adapt to the variable plant canopy architecture. These losses could potentially be mitigated through design modifications and adjustments to the machine. Particularly in *S. fruticosa* (syn. *S. triloba*), which exhibits a semi-woody and multi-branched morphology, losses are exacerbated by lower shoots remaining below the cutting line and biomass shedding (shattering) during the operation. In contrast, manual harvesting significantly minimized biomass loss by enabling a more controlled and selective cutting process. Collectively, these findings demonstrate that while mechanical harvesting offers distinct advantages in terms of labor and time efficiency, it may result in trade-offs regarding biomass recovery and harvest efficiency, leading to potential losses in yield and quality.

The fresh biomass yields obtained in the present study (5800–6700 kg ha^−1^) agree well with the ranges previously reported for S. fruticosa cultivated under Mediterranean ecological conditions, which typically vary between 5500 and 8000 kg ha^−1^ [6]. The observed variations in biomass recovery and yield can be attributed to morphological variability, a phenomenon well-documented among Greek sage populations [17]. Furthermore, the higher yield recorded in manual harvesting in this study can be explained by the reduced cutting height, which has been shown to increase harvestable biomass per unit area in perennial Lamiaceae species. Conversely, the total losses observed in mechanical harvesting fall within the 8–15% range reported in various aromatic crops, where losses are predominantly influenced by forward speed and canopy heterogeneity [8].

### 2.2. Essential Oil Content (%) and Yield (L ha^−1^)

Values are expressed as mean ± standard deviation (*n* = 3). The essential oil content and yield (L ha^−1^) of *S. fruticosa* obtained from both harvesting methods are given in Table 1. Mean comparisons between harvesting methods were performed using Tukey’s HSD test at *p* < 0.05 and independently verified by a Welch-corrected *t*-test (Table 11). The calculated least significant difference (LSD) values were 0.426 for essential oil content and 1.075 for essential oil yield, and different letters within the same column indicate statistically significant differences between harvesting methods.

Essential oil contents were determined in triplicate, and the results are presented as mean values with their corresponding standard deviations. The experimental results revealed a significant reduction in essential oil (EO) content in machine-harvested *S. fruticosa* plants (1.57%) compared to manually harvested ones (2.03%). Correspondingly, the essential oil yield was determined as 49.5 L ha^−1^ in manual harvesting, whereas it was recorded as 33.3 L ha^−1^ in mechanical harvesting. This substantial loss of approximately 22.6% can be primarily attributed to the physical rupture of fragile glandular trichomes caused by the intense vibration and friction of the reciprocating cutter bar, which leads to the immediate volatilization of sensitive monoterpenes before distillation. Furthermore, the non-selective nature of mechanical harvesting incorporates a larger proportion of woody stems compared to the selective cutting of tender, leaf-rich upper parts in manual harvesting, thereby diluting the overall oil percentage in the processed biomass. These findings suggest that while mechanization enhances operational speed, it introduces significant physical stress that compromises phytochemical integrity, aligning with reports of impact-induced losses in other *Lamiaceae* species [10]. The essential oil (EO) content obtained from manual harvesting (2.03%) falls within the typical range of 1.5–2.5% previously reported for *S. fruticosa* under Mediterranean ecological conditions [3,6,18]. This accumulation is known to be significantly influenced by various environmental and agronomic factors. The predominance of oxygenated monoterpenes such as 1,8-cineole in the present study is consistent with previous reports on Greek *S. fruticosa* populations [19]. Similar essential oil profiles characterized by high proportions of oxygenated monoterpenes, particularly 1,8-cineole and camphor, have also been reported in different *Salvia* species, including *S. fruticosa*, confirming the chemotypic consistency observed in Mediterranean sage populations [20]. However, a reduction in EO content was observed following mechanical harvesting. Such reductions in essential oil levels during mechanical handling have been attributed to the rupture of glandular trichomes and subsequent pre-distillation volatilization losses [10].

### 2.3. Essential Oil Composition (%)

**Figure 1:**
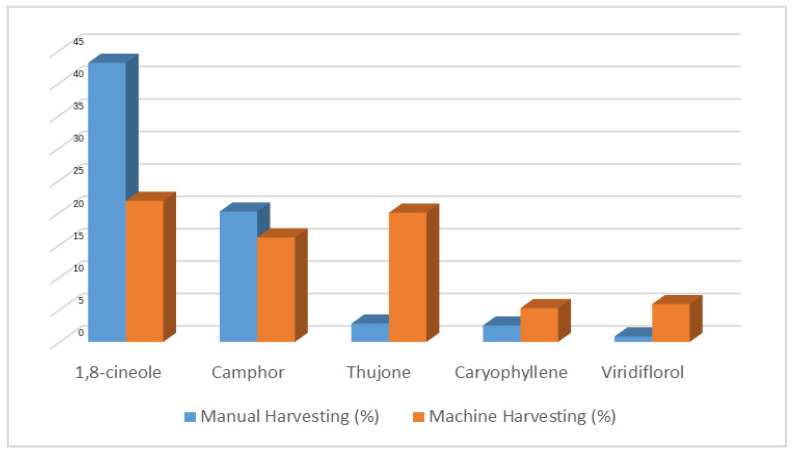
Major components of Anatolian sage oil to manual and machine harvesting.

In this study, the effects of manual and machine harvesting methods on the essential oil composition of *S. fruticosa* (syn. *S. triloba*) were comprehensively evaluated. The essential oil compositions obtained from manual and machine harvesting are given in Table 2. The major components of the essential oils obtained from different harvesting methods are also presented in Table 3. The distribution of chemical classes in *S. fruticosa* essential oil as affected by harvesting method is shown in Table 4. The major components of Anatolian sage essential oil obtained from manual and machine harvesting methods are presented in Figure 1. Essential oil constituents were analyzed by GC–MS in triplicate, and the results are presented as mean ± standard deviation (*n* = 3). Mean comparisons between harvesting methods were performed using Tukey’s honestly significant difference (HSD) test at *p* < 0.05 and independently verified by a Welch-corrected *t*-test (Table 12). The calculated least significant difference (LSD) values varied depending on the compound, generally ranging between 0.05 and 0.30 for minor components and between 0.5 and 3.5 for major components. Different letters within the same row indicate statistically significant differences between harvesting methods.

The findings demonstrate that the harvesting method significantly influences not only the component ratios but also the distribution of chemical structures and the potential biological activity.

In manually harvested samples, oxygenated monoterpenes constituted the dominant fraction of the essential oil, with particularly high concentrations of 1,8-cineole (43.07%) and camphor (20.12%). These compounds are low-molecular-weight, highly volatile structures stored in glandular trichomes. The preservation of cellular integrity and the minimization of mechanical damage to plant tissues and oil glands during manual harvesting likely facilitated the higher retention of these volatile and biologically active compounds. This preservation can be regarded as a quality indicator that enhances the therapeutic potential of the oil, particularly regarding oxygenated monoterpenes.

Conversely, a marked redistribution in the essential oil profile was observed in machine harvesting. While the proportion of 1,8-cineole decreased by nearly half (21.77%), monoterpene ketones such as thujone (19.94%) and (+)-2-bornanone (16.12%) showed a significant increase. These compositional shifts are most plausibly explained by purely physical processes occurring during and immediately after mechanical cutting: selective loss of the more volatile low-molecular-weight monoterpenes through rupture of glandular trichomes, together with a passive proportional enrichment of less volatile and heavier constituents (e.g., ketones and sesquiterpenes) within the remaining biomass. Although stress-induced alterations of terpene biosynthesis in wounded aromatic plants have been reported in the literature, such biosynthetic contributions cannot be directly demonstrated from the present dataset and are therefore mentioned here only as a hypothetical contributing mechanism rather than an established cause of the observed differences.

Moreover, the relative abundance of the sesquiterpene fraction (e.g., caryophyllene 5.22% and viridiflorol 5.84%) was found to be higher in machine-harvested samples. Sesquiterpenes are compounds with higher molecular weights and lower volatility. The proportional increase in these more stable sesquiterpenes is likely a consequence of the partial loss or volatilization of the lighter monoterpenes, shifting the chemical profile towards a “heavier” and less volatile character.

From a chemical structure perspective, manually harvested samples were characterized by a higher content of oxygenated monoterpenes, whereas mechanically harvested samples showed a predominance of ketone derivatives and sesquiterpenes. Pharmacologically, the high levels of 1,8-cineole and camphor in manually harvested material represent favorable traits due to their well-documented anti-inflammatory, mucolytic, and antimicrobial properties. In contrast, the increase in thujone content requires careful consideration regarding quality standardization because of its neuroactive properties and potential toxicity at high doses. Türkmen (2021) [20] similarly reported that *S. fruticosa* essential oil is predominantly composed of oxygenated monoterpenes, with 1,8-cineole and camphor representing the major constituents, supporting the compositional pattern observed in the present study. The predominance of 1,8-cineole and camphor in manually harvested samples is also consistent with the chemotypes of *S. fruticosa* previously reported for Mediterranean populations [3,6]. However, the compositional shifts observed in mechanically harvested plants, particularly the increase in ketone derivatives such as thujone, may reflect physiological responses associated with mechanical damage and tissue disruption during harvesting. Mechanical stress and tissue wounding in aromatic plants have been reported to influence secondary metabolism and may contribute to modifications in terpene biosynthesis [21].

It should be emphasized that the present study did not include gene expression, enzymatic activity, or jasmonate/stress-hormone measurements; consequently, any interpretation linking the observed changes to stress-induced modulation of terpene biosynthesis remains hypothetical. The comparatively higher proportions of thujone, caryophyllene, and viridiflorol in machine-harvested samples are most simply explained by differential volatility losses and by mechanical inclusion of more lignified plant fractions. The potential involvement of wound-signaling pathways previously reported in aromatic species [21,22,23,24] is acknowledged as a possible contributing factor, but verification of such mechanisms would require dedicated molecular and biochemical investigations beyond the scope of the current agronomic comparison.

To place these compound-level differences on a more conservative statistical footing, an independent Welch-corrected two-sample *t*-test was applied to the triplicate raw values of every quantifiable compound in Table 2 (24 compounds; α-thujene and cis-sabinol were not detected in the manual treatment and therefore could not be tested). Seventeen of the twenty-four compounds reached statistical significance (six at *p* < 0.001, nine at *p* < 0.01 and two at *p* < 0.05), including all five quantitatively dominant constituents (1,8-cineole, thujone, β-pinene, camphor’s oxidative partner bornyl acetate, and viridiflorol). The agreement with the parametric Tukey HSD groupings is very high across the compositional profile, with only two minor discrepancies: caryophyllene (Tukey: different letters; Welch: *p* = 0.087) and eugenol (Tukey: different letters; Welch: *p* = 0.071). In both cases the harvesting-induced change in absolute concentration is sizeable (two-fold or more), but the small sample size (*n* = 3) combined with a relatively high within-group dispersion in at least one of the groups prevents the Welch-corrected test from crossing the nominal *p* < 0.05 threshold. The Tukey groupings have been retained as originally computed, since they reflect the parametric treatment-mean comparison directly relevant to the compound-level interpretation; the Welch *p*-values are reported in Table 2 footnote and in Table 12 as an independent robustness check. Seven compounds (camphene, α-terpinolene, camphor, linalyl acetate, caryophyllene, endo-borneol and eugenol) were classified as not significantly different between the two harvesting methods, confirming that the compositional impact of the harvesting operation is concentrated on the volatile monoterpene fraction and not uniformly distributed across all essential-oil constituents.

Overall, the results indicate that the harvesting method is a determinant factor not only for yield parameters but also for the chemical composition, biological activity potential, and safety profile of the essential oil. If quality-oriented production is targeted for *S. fruticosa*, manual harvesting appears to be more advantageous for obtaining a therapeutic profile rich in oxygenated monoterpenes.

The essential oil composition of *S. fruticosa* showed clear differences in chemical class distribution depending on the harvesting method. Oxygenated monoterpenes were the dominant group in both samples, accounting for 75.06% in manually harvested plants and 69.56% in machine-harvested plants. This group mainly included 1,8-cineole, camphor, thujone, linalool, borneol derivatives, and related oxygenated compounds.

Monoterpene hydrocarbons were present at similar levels in both harvesting methods, comprising 15.73% in manual harvesting and 16.01% in machine harvesting, and were mainly represented by α-pinene, β-pinene, myrcene, limonene, and γ-terpinene.

In contrast, sesquiterpene hydrocarbons and oxygenated sesquiterpenes were found in higher proportions in machine-harvested samples. Sesquiterpene hydrocarbons increased from 3.70% (manual) to 6.70% (machine), while oxygenated sesquiterpenes showed a marked increase from 1.25% to 7.34%, largely due to higher levels of caryophyllene oxide and viridiflorol.

These results indicate that machine harvesting may promote the relative accumulation of heavier and more oxidized compounds, while manual harvesting better preserves oxygenated monoterpenes, particularly 1,8-cineole.

It should be noted that the total percentages of the classified compounds may not reach 100%, as some minor constituents were not included in the classification or were present in trace amounts and categorized as unidentified or “others”.

### 2.4. Macro and Micro Elements (ppm)

In this study, the effects of machine and manual harvesting methods on the macro and micro element composition of *S. fruticosa* (syn. *S. triloba*) were comprehensively evaluated. The macro and micro element contents obtained from manual and machine harvesting are given in Table 5 and Table 6, respectively. Values are expressed as mean ± standard deviation (*n* = 3). Mean comparisons between harvesting methods were performed using Tukey’s HSD test at *p* < 0.05 and independently verified by a Welch-corrected *t*-test (Table 11). The least significant differences (LSD) values were 4.16 (P), 48.30 (K), 104.50 (Ca), 21.60 (Mg), and 18.60 (S) for macro elements, and 4.18 (Fe), 0.74 (Zn), 0.16 (Cu), 0.42 (Mn), and 0.87 (B) for micro elements. Different letters within the same column indicate statistically significant differences between harvesting methods.

The findings indicate that the harvesting method influences not only element concentrations but also the general consistency of the measurements. Regarding macro elements, calcium (Ca) and sulfur (S) contents were significantly higher in manually harvested samples. Given the fundamental role of calcium in cell wall structure and membrane stability, it can be postulated that manual harvesting better preserves tissue integrity, while selective leaf picking ensures the inclusion of mineral-rich fractions. Calcium accumulates largely in the cell wall in the form of calcium pectate and remains stable in the tissue due to its lack of phloem mobility. Conversely, the physical impact, friction, and tissue bruising associated with mechanical harvesting may compromise cell wall integrity, leading to the loss or proportional dilution of certain minerals. Furthermore, the inclusion of stems and more fibrous tissues in the mechanical harvest likely reduces the overall mineral density.

The higher levels of potassium (K) and phosphorus (P) observed in manual harvesting can be attributed to the selection of metabolically active, leaf-dominant tissues. These elements play critical roles in energy metabolism, osmotic balance, and cellular regulation processes. In contrast, the higher magnesium (Mg) content found in machine harvesting may be explained by the incorporation of stems and older tissues into the mixture; although Mg is the central atom of the chlorophyll molecule, it can also be present in significant amounts in older tissues.

Regarding micro elements, zinc (Zn), copper (Cu), and boron (B) contents were notably higher in manual harvesting. Zn and Cu serve as cofactors in antioxidant enzyme systems and are closely linked to oxidative stress mechanisms. The superior preservation of cellular structure and tissue integrity in manual harvesting may have limited the loss of these elements. The lack of significant differences in iron (Fe) and manganese (Mn) contents between the two methods can be explained by their presence in more stable fractions within the tissue and their lower mobility.

From a human health perspective, the higher Ca content in manually harvested samples may offer potential contributions to bone mineralization, muscle contraction, and nerve transmission. Additionally, the elevated levels of Zn and Cu are significant for immune system functions, antioxidant defense mechanisms, and cellular repair processes. This suggests that manually harvested *S. fruticosa* may possess higher nutraceutical and pharmaceutical value. Overall, the findings reveal that the harvesting method is a determinant factor not only for agronomic yield but also for the chemical quality and functional properties of the plant.

The findings of this study demonstrate that the harvesting method significantly influences the mineral profile of *S. fruticosa*. The significantly higher concentrations of calcium (Ca) and sulfur (S) in manually harvested samples align with the physiological role of these elements in plant tissues. Calcium, primarily localized in the cell wall as calcium pectate, provides structural rigidity and membrane stability [25]. The higher Ca levels in manual harvesting can be attributed to the selective collection of leaf-dominant tissues, which are known to be the primary sinks for non-mobile elements like Ca, as opposed to the more lignified and mineral-poor stem fractions often included in mechanical harvesting [26].

The elevated levels of potassium (K) and phosphorus (P) in manually harvested samples further support the “leaf-dominance” hypothesis. Since K and P are highly mobile in the phloem and concentrated in metabolically active tissues, manual selection of younger, more active leaves naturally results in higher concentrations of these elements [27]. Conversely, the higher magnesium (Mg) content observed in machine harvesting may be explained by the inclusion of older leaves and stems; while Mg is mobile, it remains relatively stable in older tissues even as other elements are translocated to newer growth [28].

Regarding micro elements, the notably higher zinc (Zn), copper (Cu), and boron (B) contents in manual harvesting are significant from a nutraceutical perspective. Zn and Cu are essential cofactors for antioxidant enzymes such as superoxide dismutase (SOD), which protects the plant against oxidative stress [29,30]. The preservation of these elements in manual harvesting suggests that the lower physical impact prevents the leaching or degradation often associated with the tissue bruising and cellular rupture occurring during mechanical operations [10].

Furthermore, the absence of significant differences in iron (Fe) and manganese (Mn) concentrations suggests that these micro-nutrients are predominantly associated with structurally stable cellular fractions and enzyme complexes, rendering them less sensitive to mechanical harvesting variations [26,27,28,29,31]. Ultimately, the superior mineral profile of manually harvested *S. fruticosa* emphasizes its higher potential for use in pharmaceutical and functional food applications, where mineral density and tissue integrity are paramount.

### 2.5. DPPH Radical Scavenging Activity (DPPH)-Results

The results of this study indicate that the antioxidant activity of *S. fruticosa* remains relatively stable regardless of the harvesting method employed. No statistically significant difference was observed between harvesting methods in terms of antioxidant activity (*p* > 0.05). The DPPH radical scavenging activity values were 0.093 ± 0.001 mg TE mL^−1^ for manual harvesting and 0.096 ± 0.001 mg TE mL^−1^ for machine harvesting (Table 7). The very small difference between the two treatments suggests that the harvesting technique had no substantial effect on the overall radical scavenging capacity of the extracts.

The stability of the antioxidant potential may be related to the persistence of phenolic compounds that contribute to the antioxidant activity of *Salvia* species. Phenolic constituents such as rosmarinic acid and various flavonoids are widely recognized as the main contributors to the antioxidant properties of sage extracts [31,32]. Because these compounds are generally more stable than volatile metabolites, the antioxidant capacity of the extracts may remain largely unaffected by differences in harvesting technique.

Welch two-sample *t*-tests conducted on the triplicate raw values of the four antioxidant endpoints (DPPH, FRAP, TPC and TFC) returned |t| values of 0.36, 2.17, 0.69 and 0.87, all classified as non-significant (*p* > 0.05; Table 5). The PCA loadings additionally show that these four variables contribute only marginally to the separation along PC1 (|loading| < 0.12), confirming that the antioxidant-related phytochemistry is largely insensitive to the harvesting method under the conditions of the present one-year trial. We therefore refrain from proposing biosynthetic or redox-signaling mechanisms that are not supported by direct measurement.

The comparable DPPH values obtained from manual and machine harvesting indicate that the radical-scavenging capacity of *S. fruticosa* extracts is largely independent of the harvesting technique. This stability most likely reflects the chemical robustness of the phenolic fraction (notably rosmarinic acid and related polyphenols), which is less susceptible to harvesting-induced losses than the volatile terpenoid fraction [31,32,33]. Reported DPPH values for *Salvia* spp. and related aromatic Lamiaceae (e.g., *Thymus vulgaris*) vary considerably with extraction solvent, developmental stage, geographical origin, and pre-harvest cultivation practice [34,35,36,37]; the values recorded here fall within the expected range for the species. From an industrial perspective, this finding supports the adoption of mechanized harvesting without significant compromise of the antioxidant-related phytochemical quality of the raw material.

### 2.6. Ferric Reducing Antioxidant Power-Results

Values are expressed as mean ± standard deviation (*n* = 3). Mean comparisons between harvesting methods were performed using Tukey’s HSD test at *p* < 0.05 and independently verified by a Welch-corrected *t*-test (Table 11). The least significant difference (LSD) value was 6.33. Different letters indicate statistically significant differences between harvesting methods.

The ferric-reducing antioxidant power (FRAP) values differed between harvesting methods, with manually harvested samples exhibiting a higher reducing capacity (382 µmol Fe^2+^ g^−1^ extract) compared with machine-harvested samples (336 µmol Fe^2+^ g^−1^ extract) (Table 8). This numerical 12% reduction, however, was not statistically significant (Welch *t*-test, *p* > 0.05; Table 11), indicating that FRAP values remain largely stable between harvesting methods. The FRAP assay reflects the overall electron-donating capacity of antioxidant compounds, mainly phenolics and flavonoids, which reduce the Fe^3+^–TPTZ complex to Fe^2+^ under acidic conditions [38]. Machine harvesting can expose plant tissues to increased physical stress, including impact, vibration, and friction, which may accelerate oxidative reactions and promote partial degradation of phenolic antioxidants prior to extraction. Similar observations have been reported in medicinal plants where mechanical damage during harvesting or postharvest handling altered antioxidant capacity and phenolic stability [39].

The FRAP values obtained in the present study fall within the range reported for *Salvia* species in the literature. For example, Loizzo et al. (2014) [40] reported FRAP values between approximately 196 and 422 µmol Fe^2+^ g^−1^ extract in different *Salvia* species, indicating that the antioxidant capacity observed in this study is consistent with previously published data. In *S. fruticosa*, antioxidant activity has also been shown to vary depending on extraction conditions, geographical origin, and phenolic composition [41]. Therefore, the moderate decrease observed under mechanical harvesting conditions may reflect subtle alterations in the phenolic antioxidant system rather than a major change in overall phytochemical composition. The ferric-reducing antioxidant power observed in the present study can largely be attributed to the electron-donating ability of phenolic constituents present in *Salvia* extracts. Previous studies have demonstrated that antioxidant capacity in Lamiaceae herbs is strongly associated with phenolic compounds that contribute to the reducing power of plant extracts [38].

### 2.7. Total Phenolic Content (TPC)-Results

Harvesting method had no statistically significant effect on total phenolic content (Welch *t*-test, *p* > 0.05; Table 11). Values are expressed as mean ± standard deviation (*n* = 3). Mean comparisons between harvesting methods were performed using Tukey’s HSD test at *p* < 0.05 and independently verified by a Welch-corrected *t*-test (Table 11). The least significant difference (LSD) value was 4.50. Since the Welch-corrected *t*-test returned *p* > 0.05, the two harvesting methods share the same letter in Table 9, indicating no statistically significant difference. Total phenolic content (TPC) showed only slight variation between harvesting methods, indicating that the overall phenolic pool of *S. fruticosa* was largely preserved under both harvesting conditions. In the present study, manually harvested samples exhibited a slightly higher TPC value (134.6 ± 2.12 mg GAE g^−1^ extract) compared with machine-harvested samples (129.3 ± 1.85 mg GAE g^−1^ extract). Phenolic compounds represent one of the major antioxidant groups in *Salvia* species and play a key role in the biological activity of plant extracts. The relatively small difference observed between manual and mechanical harvesting suggests that phenolic accumulation in *S. fruticosa* is primarily controlled by genetic and environmental factors rather than by harvesting technique alone. Similar findings have been reported in several *Salvia* species where total phenolic content remained relatively stable despite variations in cultivation or processing conditions.

Total phenolic content represents an important indicator of the antioxidant potential of *Salvia* species, as phenolic acids and related polyphenols constitute a major fraction of the secondary metabolites responsible for biological activity in sage extracts [42]. Previous studies have indicated that TPC values in *Salvia* extracts may vary considerably depending on extraction solvent, geographical origin, and plant developmental stage [40,41,43]. Therefore, the values obtained in the present study fall within the expected range reported for *Salvia* species and indicate that harvesting method has only a limited influence on the overall phenolic content of *S. fruticosa*.

### 2.8. Total Flavonoid Content (TFC)-Results

Harvesting method did not have a statistically significant effect on total flavonoid content (*p* > 0.05). Total flavonoid content (TFC) exhibited moderate variation between harvesting methods. In the present study, machine-harvested samples showed slightly higher TFC values (25.2 ± 1.48 mg QE g^−1^ extract) compared with manually harvested samples (22.7 ± 1.34 mg QE g^−1^ extract) (Table 10). Flavonoids constitute an important subclass of phenolic metabolites and contribute significantly to the antioxidant properties of *Salvia* extracts through their strong electron-donating and radical-scavenging activities. Variations in TFC between harvesting treatments may be related to differences in tissue integrity and exposure of plant material to environmental factors such as oxygen and light during the harvesting process. Machine harvesting may increase tissue disruption and surface exposure, which could influence the stability or extraction efficiency of certain flavonoid compounds.

The TFC values obtained are consistent with the ranges reported for *Salvia* species [40,41,44] and indicate that, although harvesting method may cause limited variation in individual flavonoids, the overall flavonoid profile of *S. fruticosa* extracts remains largely stable.

### 2.9. Some Operational Characteristics of Machine and Manual Harvesting

During the studies, the machine forward speed was determined as 3 km h^−1^, and the average machine work efficiency at this speed was calculated as 0.315 ha h^−1^. The calculated work efficiency may vary depending on the idle time.

It was determined that the hourly work efficiency of a worker ranged between 0.0114–0.0138 ha h^−1^. The worker efficiency varies depending on plant density and the transport distance of the harvested plants.

Machine work efficiency was determined to be approximately 25 times that of human work efficiency. In other words, the area harvested by a machine can be harvested by eight workers in the same amount of time. While 1 ha of land can be harvested in approximately 3.18 h with the machine, it can be harvested in 80 h with a worker. Considering the difficulty of accessing labor and the high cost of labor, especially in the harvesting of large areas, the necessity of harvesting with a machine is understood.

Substantial increases in effective field capacity are commonly observed in mechanized harvesting systems due to reduced labor dependency and higher operational continuity. In contrast, manual harvesting performance is inherently variable and strongly affected by plant density, field layout, and handling logistics. From a machinery management perspective, effective field capacity is primarily influenced by forward speed, working width, and field efficiency parameters [45].

Given the increasing constraints in accessing seasonal agricultural labor and the rising costs of manual work, the transition toward mechanized systems is widely recognized as a key factor for economic sustainability in crop production systems [40]. These findings indicate that while manual harvesting may remain suitable for small-scale, high-value production, mechanized harvesting offers clear advantages in meeting large-scale market demand and maintaining competitive production efficiency.

### 2.10. Multivariate Analysis of the Compositional Response

**Figure 2:**
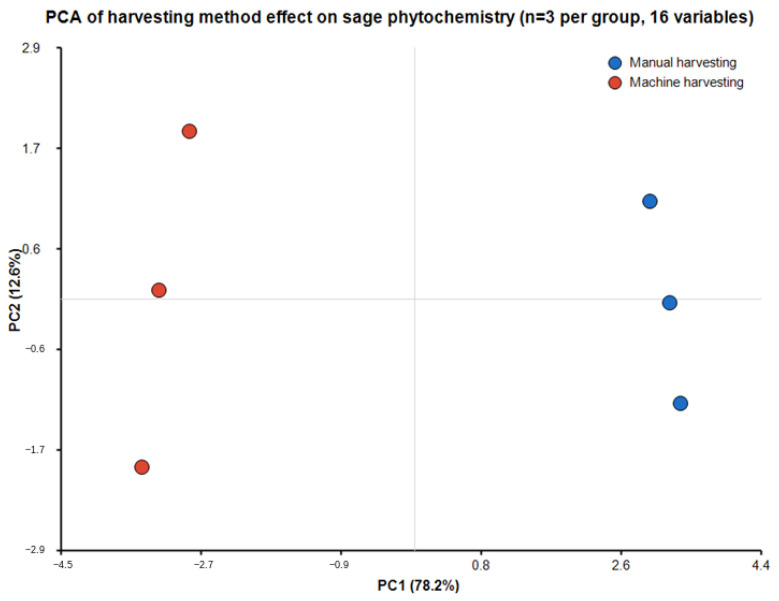
Principal component analysis (PCA) score plot based on the standardized raw triplicate values of sixteen quantitative parameters measured on the manual- and machine-harvested *Salvia fruticosa* samples (*n* = 3 per group). PC1 accounts for 78.2% of the total variance and PC2 for 12.7%, giving a cumulative explained variance of 90.9%. Complete separation between harvesting groups along PC1 confirms that the harvesting method produces a coherent, multivariate compositional shift rather than isolated univariate differences.

To provide a comprehensive, integrated view of the sixteen quantitative parameters measured on both harvesting groups (essential-oil content and yield, ten mineral elements, and four antioxidant indicators), a principal component analysis (PCA) was performed on the standardized raw triplicate data (*n* = 3 per group). The first two principal components jointly explained 90.9% of the total variance (PC1 = 78.2%; PC2 = 12.7%), indicating that a two-dimensional projection faithfully represents the overall response profile. As shown in Figure 2, the three manual-harvested samples clustered tightly at positive PC1 scores while the three machine-harvested samples clustered at negative PC1 scores, yielding a clean, non-overlapping separation along the dominant axis. This pattern demonstrates that the differences between harvesting methods are not restricted to individual parameters but manifest as a coherent, multivariate shift in the chemical signature of the raw material.

**Figure 3:**
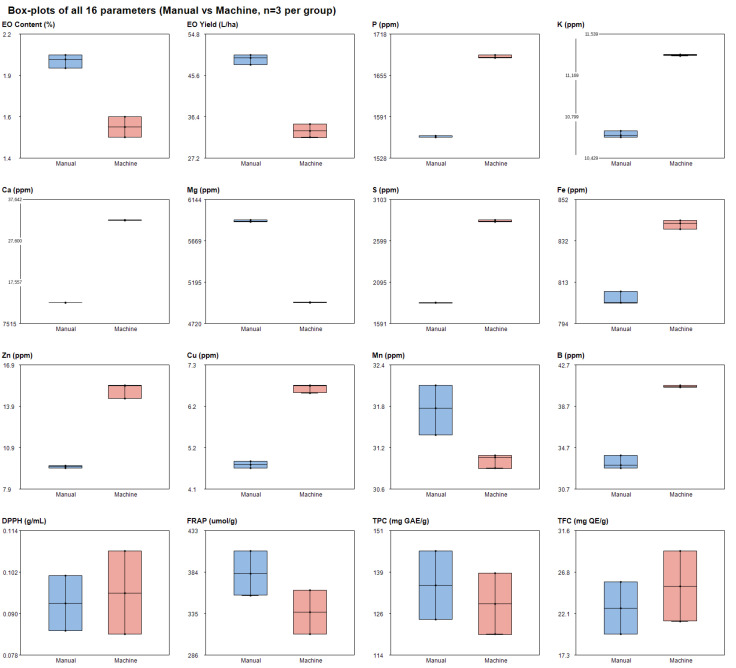
Box-plot matrix (4 × 4) of all sixteen measured parameters for the manual- and machine-harvested *Salvia fruticosa* samples (*n* = 3 per group). Rows 1–2 show essential-oil content and yield and the macro/micro-nutrients P, K, Ca, Mg, S, Fe; rows 3–4 show the remaining micro-nutrients Zn, Cu, Mn, B and the antioxidant indicators DPPH, FRAP, TPC and TFC. Boxes span the data range, the central horizontal line represents the triplicate median, and individual points mark the three replicate values.

To complement the PCA and provide a transparent visual summary of the parameter-level variation, the triplicate raw values of all sixteen response variables (essential-oil content and yield, the ten mineral elements P, K, Ca, Mg, S, Fe, Zn, Cu, Mn and B, and the four antioxidant indicators DPPH, FRAP, TPC and TFC) are displayed as a 4 × 4 box-plot matrix in Figure 3. This panel view allows direct side-by-side inspection of the manual and machine groups for every measured variable and visually corroborates the pair-wise Welch *t*-tests reported in Table 11; the corresponding compound-level Welch *t*-test results for the 24 essential-oil constituents are presented in Table 12.

**Table 11:** Welch two-sample *t*-test results comparing raw triplicate measurements between manual and machine harvesting (*n* = 3 per group). Mean +/− standard deviation, |t| statistic, approximate degrees of freedom, and *p*-value class are reported for every quantitative parameter.

Parameter	Manual (Mean +/− SD)	Machine (Mean +/− SD)	|t|	*p*
EO Content (%)	2.02 +/− 0.05	1.57 +/− 0.07	9.71	<0.01
EO Yield (L ha^−1^)	49.23 +/− 1.13	33.30 +/− 1.50	14.79	<0.001
P (ppm)	1683.13 +/− 2.66	1560.66 +/− 1.37	70.90	<0.001
K (ppm)	11,350.17 +/− 5.83	10,640.23 +/− 29.30	41.10	<0.001
Ca (ppm)	32,564.92 +/− 57.75	12,573.18 +/− 32.31	524.0	<0.001
Mg (ppm)	4960.92 +/− 4.18	5894.43 +/− 11.16	135.9	<0.001
S (ppm)	2838.68 +/− 11.75	1845.32 +/− 2.26	143.6	<0.001
Fe (ppm)	840.28 +/− 2.09	805.52 +/− 3.03	16.35	<0.001
Zn (ppm)	15.12 +/− 0.54	9.53 +/− 0.10	17.69	<0.001
Cu (ppm)	6.69 +/− 0.11	4.72 +/− 0.09	24.20	<0.001
Mn (ppm)	30.98 +/− 0.10	31.71 +/− 0.36	3.40	<0.05
B (ppm)	40.58 +/− 0.09	33.20 +/− 0.66	19.18	<0.001
DPPH (g mL^−1^)	0.0930 +/− 0.0080	0.0960 +/− 0.0120	0.36	n.s.
FRAP (umol g^−1^)	382.0 +/− 26.00	336.0 +/− 26.00	2.17	n.s.
TPC (mg GAE g^−1^)	134.6 +/− 10.00	129.3 +/− 9.00	0.69	n.s.
TFC (mg QE g^−1^)	22.70 +/− 3.00	25.20 +/− 4.00	0.87	n.s.

Note: n.s., not significant (*p* > 0.05).

**Table 12:** Welch two-sample *t*-test results for the 24 quantifiable essential-oil compounds of Table 2 (manual vs. machine harvesting, *n* = 3 per group). Mean +/− standard deviation, |t| statistic, approximate Welch-Satterthwaite degrees of freedom, and *p*-value class are reported for every compound.

Compound	Manual (Mean +/− SD)	Machine (Mean +/− SD)	|t|	*p*
α-pinene	2.77 +/− 0.12	2.50 +/− 0.09	3.12	<0.05
Camphene	2.57 +/− 0.11	2.50 +/− 0.08	0.89	n.s.
β-pinene	4.03 +/− 0.15	2.69 +/− 0.10	12.87	<0.001
Myrcene	2.13 +/− 0.08	1.35 +/− 0.06	13.51	<0.001
Limonene	1.64 +/− 0.07	2.20 +/− 0.09	8.51	<0.01
1,8-cineole	43.07 +/− 0.85	21.77 +/− 3.48	10.30	<0.01
γ-terpinene	0.57 +/− 0.04	0.85 +/− 0.05	7.57	<0.01
p-cymene	0.29 +/− 0.02	1.75 +/− 0.07	34.74	<0.001
α-terpinolene	0.48 +/− 0.03	0.43 +/− 0.02	2.40	n.s.
Thujone	2.83 +/− 1.94	19.94 +/− 0.76	14.22	<0.01
Camphor	20.12 +/− 2.47	16.12 +/− 4.73	1.30	n.s.
Linalool	0.55 +/− 0.04	0.33 +/− 0.02	8.52	<0.01
Linalyl acetate	0.85 +/− 0.05	0.88 +/− 0.04	0.81	n.s.
Bornyl acetate	1.68 +/− 0.08	1.30 +/− 0.06	6.58	<0.01
Caryophyllene	2.54 +/− 0.04	5.22 +/− 1.47	3.16	n.s.
Humulene	1.16 +/− 0.04	1.36 +/− 0.07	4.30	<0.05
(+)-4-Carene	1.25 +/− 0.07	0.29 +/− 0.02	22.84	<0.001
Endo-borneol	0.99 +/− 0.05	0.96 +/− 0.04	0.81	n.s.
Geranyl acetate	0.14 +/− 0.01	0.05 +/− 0.01	11.02	<0.001
Geraniol	0.11 +/− 0.01	0.06 +/− 0.01	6.12	<0.01
Caryophyllene oxide	0.40 +/− 0.03	1.50 +/− 0.06	28.40	<0.001
Viridiflorol	0.85 +/− 0.08	5.84 +/− 0.44	19.33	<0.01
Thymol	0.03 +/− 0.02	0.13 +/− 0.01	7.75	<0.01
Eugenol	0.02 +/− 0.01	0.04 +/− 0.01	2.45	n.s.

Note: Welch *t*-test. n.s., *p* > 0.05. α-thujene, cis-sabinol undetected in manual.

A particularly noteworthy observation that becomes explicit in the multivariate display is the behavior of the mineral profile across the two harvesting groups. Calcium concentration decreased from 32,564.92 +/− 57.75 ppm in the manually harvested samples to 12,573.18 +/− 32.31 ppm in the machine-harvested samples, a 2.6-fold reduction that is statistically highly significant (Welch *t*-test, *p* < 0.001). More moderate but consistent reductions were recorded for zinc (−37%, *p* < 0.001), copper (−29%, *p* < 0.001), boron (−18%, *p* < 0.001) and sulphur (−35%, *p* < 0.001), whereas iron showed only a minor decrease (−4.1%, *p* < 0.001). Conversely, potassium, phosphorus and magnesium were slightly higher in the machine-harvested biomass. This pattern indicates that the lower cutting height and selective collection of leaf-dominant tissues in manual harvesting preserve a mineral-rich fraction, whereas non-selective mechanical cutting incorporates a larger proportion of stems and lignified tissues, which are depleted in non-mobile leaf-sink elements such as calcium, zinc and copper. The calcium, zinc, copper, boron and sulphur panels in Figure 3 visually confirm the magnitude and the remarkable consistency of this effect across the triplicates. From a production standpoint, this finding is operationally important because the mineral fraction of the raw material contributes to its nutritional and pharmaceutical value, and the lower mineral density observed under mechanical harvesting should be considered when the biomass is intended for herbal or functional-food applications.

### 2.11. Limitations of the Study

Several limitations of the present work should be stated explicitly, as they delimit the scope within which the conclusions can be interpreted. First, the study is based on a single growing season (2024) and on a single trial site in Konya Province, so the absolute values reported here cannot be generalized across years or across the wider Mediterranean distribution range of *Salvia fruticosa*, because phytochemical composition in aromatic plants has been shown to vary markedly even within a single taxon at the edges of its distribution range [46,47]. Essential oil yield and antioxidant capacity in *Salvia* species are known to be strongly modulated by interannual variation in temperature, rainfall and photoperiod [36,48], and the 2024 season in central Anatolia was in fact characterized by a 0.8 °C positive thermal anomaly and a 14% overall precipitation deficit relative to the 1994–2023 long-term average, with a particularly marked April–May rainfall shortfall (Table 13). The quantitative outcomes reported here should therefore be viewed as representative of a moderately warm and dry season and not as a long-term mean. Second, each treatment was replicated three times (*n* = 3), which is appropriate for pair-wise mean comparison under the controlled agronomic design but limits the statistical power of inferential multivariate procedures; accordingly, the principal component analysis presented in Section 2.4. is used exclusively in a descriptive sense to visualize treatment structure rather than to test hypotheses. Third, a formal year × harvesting-method interaction term could not be fitted, because the year factor has only one level. A multi-year, multi-site follow-up, preferably coupled with parallel monitoring of soil moisture and air temperature during the pre-flowering window, is therefore required to quantify the interannual stability of the harvesting-method effect, in particular for the antioxidant endpoints (DPPH, FRAP, TPC, TFC), whose season-to-season fluctuation in *Salvia* species has been reported to be considerable. Finally, the mineral composition differences between harvesting methods were inferred from ICP measurements on the herbage only; complementary tissue-fraction analyses (separate ICP measurements on leaves, stems and lignified fractions) would be required in future work to quantify precisely the stem-to-leaf ratio contribution to the patterns reported here, which is beyond the scope of the present agronomic trial and is proposed as the next methodological step.

## 3. Materials and Methods

### 3.1. Trial Area and Plant Material

**Figure 4:**
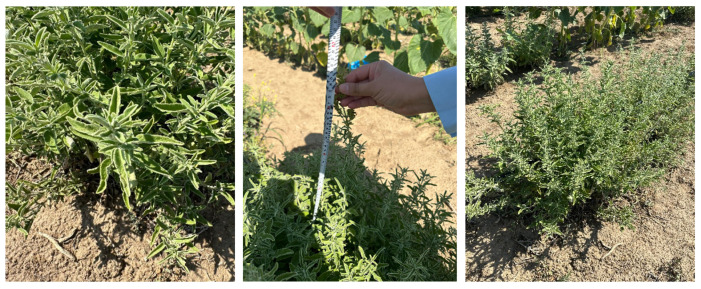
Pictures of Anatolian sage (*S. fruticosa*).

The field experiments were carried out during the 2024 growing season at a dedicated research site in the vicinity of Göztepe, Karaman, Türkiye (37.18° N, 33.22° E; ~1050 m a.s.l.). The region is characterized by a semi-arid continental climate with high solar irradiance, low relative humidity, and pronounced diurnal temperature fluctuations, conditions that are highly conducive to secondary metabolite accumulation in aromatic species. The soil at the experimental site exhibited a clay-loam texture with low organic matter content and a slightly alkaline reaction (pH~7.8). Because the present experiment was conducted during a single growing season, the results reported herein should be regarded as preliminary, and interannual (year-to-year) variability could not be evaluated within the scope of this work. *S. fruticosa* Mill. (syn. *S. Triloba* L.) was harvested at the full flowering stage on 15 July 2024. Images of Anatolian sage are given in Figure 4.

The plant material was botanically identified as *Salvia fruticosa* Mill. (syn. *S. triloba* L.) in accordance with the Turkish Plants Data Service (TUBIVES; http://194.27.225.161/yasin/tubives/index.php?sayfa=1&tax_id=8036/ (accessed on 17 April 2026)) and verified at the Department of Field Crops, Division of Medicinal Plants, Faculty of Agriculture, Selçuk University. No registered commercial cultivar of *S. fruticosa* exists in Türkiye; therefore, the experimental plants were established from seedlings commercially produced by Uludağ Agro A.Ş. (Bursa, Türkiye), which had been propagated from domesticated Turkish material rather than from a registered variety.

The climatic conditions of the 2024 growing season at the Göztepe-Karaman experimental site, together with the 1994–2023 long-term average of the nearest Turkish State Meteorological Service station (Karaman; WMO code 17246), are summarized in Table 13. The 2024 season was on average 0.8 °C warmer and 14% drier than the 30-year reference period, with a particularly marked precipitation deficit during the critical April-May pre-flowering period (−38%). These meteorological conditions are acknowledged as a factor that may have influenced the absolute values of essential-oil yield and of some secondary metabolites reported here, and that limits the direct generalization of the present single-season results to other years.

### 3.2. Harvesting Methods

The study focused on a perennial Lamiaceae species, Anatolian sage (*S. triloba* L.; syn. *S. fruticosa* Mill “The experimental site was established by a commercial grower for essential oil production. The plantation, established in 2022, covers a total area of 1.2 hectares and includes *S. fruticosa* Mill. The S. fruticosa plants were established with an inter-row spacing of 1500 mm and an intra-row spacing of 600 mm.

To ensure phytochemical consistency across treatments, *S. fruticosa* plants were harvested at the full flowering stage corresponding to phenological stage 65 on the BBCH scale. This harvest timing was selected to capture the period when essential oils and bioactive compounds reach their highest concentrations within the glandular trichomes [49].

#### 3.2.1. Machine Harvesting

Machine harvesting was performed using a tractor-mounted medicinal and aromatic plant harvesting machine powered by a hydraulic motor system. The machine had an adjustable cutting width ranging between 0.50 and 1.50 m. The power transmission was provided through a PTO system operating at 540 rpm, and harvesting was carried out at a forward speed of 3 km h^−1^. Cutting height and working depth were kept constant across all plots. The harvested biomass from each plot was collected and weighed separately.

#### 3.2.2. Manual Harvesting

Manual harvesting was performed using a traditional sickle. To ensure comparability between the methods, plants were manually cut at the same height as the mechanical harvester. Harvested plant material was immediately weighed to determine fresh biomass.

### 3.3. Determination of Some Pre- and Post-Harvest Physical Characteristics

Plant height was measured at the full flowering stage during harvest by recording the distance from the soil surface to the highest vegetative point on randomly selected plants within each plot, excluding border rows. Plant diameter was determined based on the widest canopy width pre-harvest and repeated on the same plants post-harvest to evaluate the effect of the harvesting mechanism on plant morphology. Cutting height was determined by measuring the distance between the soil surface and the cutting point after harvest.

To estimate harvest losses, the number of shattered plants and uncut plants, along with the total tillering count, were recorded for each plot. The harvest loss percentage was calculated using the following formula [50]:

Harvest Loss (%) = [(Number of Uncut Plants + Number of Shattered Plants)/Total Tillering Count] × 100

Fresh weight per plant was determined by weighing harvested plants, and samples were dried at 65 °C until constant weight to determine dry weight per plant. Herbage yield was calculated by weighing the total fresh biomass obtained from each plot and converting the values to an area basis (kg ha^−1^), while dry herbage yield was determined using dry matter content. All morphological measurements were conducted on a minimum of five plants per plot according to standard procedures [51,52].

### 3.4. Determination of Essential Oil Content and Yield

The essential oil content was isolated from the dried aerial parts of the plants via hydrodistillation using a Clevenger-type apparatus. The distillation process was conducted for a duration of 3 h, strictly following the protocols described in the European Pharmacopoeia [53]. The obtained essential oil volume was read directly from the graduated tube, and the content was expressed as volume per dry weight (*v*/*w*; mL 100 g). GC–MS analysis was performed to determine the essential oil composition. The essential oils were stored at −200 °C until analyzed. To determine the essential oil yield per hectare (L ha^−1^), the essential oil content ratio was extrapolated by multiplying it with the total dry herbage yield values obtained from the respective plots.

### 3.5. Essential Oil Composition

Essential oil composition was determined by gas chromatography–mass spectrometry (GC–MS) using an Agilent 7890B GC system coupled with an Agilent 5977A MSD mass selective detector (Agilent Technologies, Santa Clara, CA, USA). Separation was performed on a DB-Waxetr capillary column (60 m × 0.25 mm i.d., 0.25 μm film thickness). The oven temperature program was as follows: the column temperature was initially held at 60 °C for 10 min, then increased to 220 °C at a rate of 4 °C min^−1^ and subsequently raised to 240 °C at 10 °C min^−1^. The injector temperature was set at 250 °C. Helium was used as the carrier gas with an inlet pressure of 9.60 psi and a linear velocity of 7 cm s^−1^. The initial flow rate was 0.3 mL min^−1^. Samples (1.0 μL) were injected in split mode (split ratio 65:1). Mass spectra were acquired in electron-ionization (EI) mode at 70 eV with the ion source maintained at 230 °C and the MS transfer line held at 280 °C. Spectra were recorded in full-scan mode over the mass range m/z 40–400 at a scan rate of 2.91 scans s^−1^, with a solvent delay of 3 min.

Identification of essential oil components was based on comparison of their mass spectra with those stored in commercial libraries, including the NIST/EPA/NIH Mass Spectral Library, Wiley GC–MS Library, Adams Library, and MassFinder 3 Library, as well as with published MS data. In addition, Kovats retention indices (RI) were calculated relative to a homologous series of n-alkanes (C8–C30) analyzed under the same chromatographic conditions and compared with literature RI values for confirmation of compound identification [53].

To strengthen the identification of the most abundant constituents, the eight major compounds of the essential oil profile—1,8-cineole, alpha-pinene, beta-pinene, camphor, alpha-thujone, beta-thujone, borneol and caryophyllene oxide—were additionally confirmed by co-injection of analytical-grade authentic standards (all purity ≥ 98%, Sigma-Aldrich, St. Louis, MO, USA) analyzed under identical chromatographic conditions. Co-elution within +/− 0.02 min and superimposable EI mass spectra (match factor ≥ 900/1000) were required for confirmation. For minor constituents (relative peak area < 1%), identification relied on library matching and RI comparison only and is therefore reported as tentative. A library match factor of ≥850 was accepted as the minimum threshold for all reported compounds. It should be explicitly stated that the compositional data in Table 3 correspond to relative peak-area percentages normalized to 100% and are therefore semi-quantitative: no response-factor calibration was performed for individual components, and absolute concentrations (mg g^−1^ of essential oil) were not determined. This approach is standard in comparative essential oil profiling but cannot be used to derive absolute quantitative conclusions for individual compounds.

The retention indices were calculated using the following equation:

$$RI=100\times \left[n+\frac{\left(t_{r}(x)-t_{r}(n)\right)}{\left(t_{r}(n+1)-t_{r}(n)\right)}\right]$$

where $t_{r}\left(x\right)$ was the retention time of the compound, $t_{r}\left(n\right)$ and $t_{r}\left(n+1\right)$ were the retention times of the n-alkanes eluting immediately before and after the compound, respectively, and n is the carbon number of the preceding n-alkane.

Authentic reference standards were not available for each of the individual constituents; therefore, compound identification relied on the combined evaluation of mass-spectral matching against the above-mentioned commercial libraries and the comparison of experimentally determined Kovats retention indices with published values. Accordingly, the compositional percentages reported in this study should be considered as semi-quantitative relative abundances obtained from GC–MS peak-area normalization, rather than absolute concentrations determined against external calibration standards.

### 3.6. Determination of Macro and Micro Elements

The macro and micro elements of *S. fruticosa* herb were determined by inductively coupled plasma mass spectrometry (ICP-MS) following the NMKL Method No. 186. Approximately 0.5 g of dried and ground sample was subjected to microwave-assisted acid digestion using concentrated nitric acid (HNO_3_). The digested solutions were diluted with ultrapure water and analyzed by ICP-MS. Elemental concentrations were calculated based on external calibration curves prepared using certified standard solutions. Results are expressed in mg/kg (ppm) of dry weight. The method provides high sensitivity and accuracy for multi-elemental analysis in food matrices [54].

### 3.7. Preparation of Plant Extracts

The dried plant samples were ground into a fine powder. For the extraction process, 10 g of the powdered plant material was accurately weighed and mixed with 100 mL of ethanol. The mixture was agitated at room temperature to ensure efficient extraction of bioactive compounds. The resulting extract was then filtered through Whatman No. 1 filter paper to remove solid residues. The clear filtrates were stored at 4 °C until the antioxidant analysis was performed [53].

### 3.8. Antioxidant Activity

Antioxidant activity was determined using the DPPH radical scavenging method described by Brand-Williams et al. (1995) [55]. First, a standard Trolox stock solution was prepared in methanol, and a calibration curve was constructed using varying concentrations of this standard. For the assay, 4 mL of a 0.004% (*w*/*v*) DPPH (1,1-diphenyl-2-picrylhydrazyl) solution was added to the plant extracts and Trolox standards. The samples were incubated in the dark at room temperature for 30 min, after which the absorbance values were measured spectrophotometrically at 517 nm. The DPPH radical scavenging activity was calculated using the linear equation of the calibration curve and expressed as Trolox equivalents (mg TE mL^−1^).

### 3.9. Ferric Reducing Antioxidant Power

Ferric-reducing antioxidant power (FRAP) was determined according to the method described by Benzie and Strain (1996) [38] with slight modifications. The FRAP reagent was freshly prepared by mixing acetate buffer (300 mM, pH 3.6), TPTZ solution (10 mM in 40 mM HCl), and FeCl_3_·6H_2_O solution (20 mM) in a ratio of 10:1:1. An aliquot of 100 µL of plant extract was mixed with 3 mL of FRAP reagent and incubated at 37 °C for 10 min. Absorbance was measured at 593 nm. The results were expressed as µmol Fe^2+^ equivalents per g extract.

### 3.10. Total Phenolic Content (TPC)

Total phenolic content was determined using the Folin–Ciocalteu method according to Singleton and Rossi (1965) [15] with minor modifications. Briefly, 0.5 mL of plant extract was mixed with 2.5 mL of 10% Folin–Ciocalteu reagent. After 5 min, 2 mL of 7.5% sodium carbonate solution was added and the mixture was incubated in the dark for 30 min at room temperature. Absorbance was measured at 765 nm using a UV–Vis spectrophotometer. Gallic acid was used as the calibration standard, and the results were expressed as mg gallic acid equivalents (GAE) per g extract.

### 3.11. Total Flavonoid Content (TFC)

Total flavonoid content was determined using the aluminum chloride colorimetric method described by Chang et al. (2002) [41]. Briefly, 0.5 mL of plant extract was mixed with 0.1 mL of 10% aluminum chloride, 0.1 mL of potassium acetate (1 M), and 4.3 mL of distilled water. After incubation at room temperature for 30 min, absorbance was measured at 415 nm. Quercetin was used as the reference compound, and the results were expressed as mg quercetin equivalents (QE) per g extract.

### 3.12. Determination of Some Operational Characteristics of Machine and Manual Harvesting

Machine work efficiency: During harvesting, the operational performance parameters of the machine were determined and calculated according to the following formula [50]:

S_a_ = 0.1.B.V. K

S_a_: Machine field capacity (ha h^−1^);

B: Working width (m);

V: Forward speed of the machine (km h^−1^);

K: Field efficiency coefficient (%).

Labor (worker) field capacity: During manual harvesting, the time required for a single worker to harvest one plant and a defined group of plants within a specified distance was recorded. Harvest duration was determined accordingly, and the harvested area within that time was converted to a unit area basis (ha).

### 3.13. Statistical Analysis and Data Evaluation

All experiments were conducted in triplicate, and the results are presented as mean ± standard deviation (SD). Statistical analyses were performed using JMP software (JMP Pro, Version 19, SAS Institute Inc., Cary, NC, USA). Data were subjected to Tukey’s honestly significant difference (HSD) test at *p* < 0.05 to determine differences among means, and an independent samples *t*-test was applied to compare harvesting methods. The least significant difference (LSD) values were also calculated. Differences were considered statistically significant at *p* < 0.05, and different letters within the same column indicate significant differences between means. It should be noted that all analyses were performed with three biological replicates per treatment (*n* = 3), which is adequate for Tukey’s HSD comparison of means under controlled agronomic conditions but represents a limited sample size for inferential multivariate statistics. For this reason, the multivariate structure of the essential oil dataset was additionally inspected by principal component analysis (PCA) at the compound level, and the results are presented only in a descriptive manner. Furthermore, since the experiment was conducted during a single growing season (2024), the year factor could not be incorporated into the statistical model, and the reported outcomes should therefore be interpreted as preliminary single-season results requiring confirmation under multi-year conditions.

## 4. Conclusions

Within the limits of a single-year, single-site experiment conducted during the 2024 growing season in Central Anatolia, this study demonstrates that the harvesting method in *S. fruticosa* (syn. *S. triloba*) represents not only an agronomic operation but also a critical factor influencing phytochemical quality and production efficiency. Machine harvesting caused partial structural disruption of plant tissues and glandular trichomes, leading to noticeable alterations in essential oil composition. In particular, the proportion of the major compound 1,8-cineole decreased from 43.07% in manual harvesting to 21.77% in machine harvesting, indicating that volatile terpenoid constituents are highly sensitive to mechanical impacts during harvesting. In addition, higher harvest losses and a reduction in biomass yield were observed under machine harvesting conditions, mainly due to differences in cutting height and plant handling during the harvesting process.

In contrast to the volatile fraction, antioxidant-related parameters showed only minor variation between harvesting treatments. DPPH radical scavenging activity remained very similar between manual (0.093 mg TE mL^−1^) and machine harvesting (0.096 mg TE mL^−1^). Likewise, total phenolic content (TPC) values were 134.6 mg GAE g^−1^ extract in manual harvesting and 129.3 mg GAE g^−1^ extract in machine harvesting, indicating that the overall phenolic pool was largely preserved. Total flavonoid content (TFC) showed slightly higher values under machine harvesting (25.2 mg QE g^−1^ extract) compared with manual harvesting (22.7 mg QE g^−1^ extract), while FRAP values were 382 and 336 µmol Fe^2+^ g^−1^ extract for manual and machine harvesting, respectively. These results suggest that although machine harvesting may influence certain antioxidant-related parameters, the phenolic antioxidant system of *S. fruticosa* remains relatively stable compared with the more sensitive volatile terpenoid fraction.

From a production perspective, the substantial increase in operational efficiency achieved through machine harvesting represents a significant advantage for large-scale cultivation systems. While manual harvesting may remain feasible in small-scale production, machine harvesting appears essential for sustainable and economically viable sage production, particularly under the semi-arid continental ecological conditions of Central Anatolia. Therefore, optimizing mechanization parameters based on scientific evidence could reduce quality losses associated with machine harvesting and help achieve phytochemical profiles closer to those obtained through manual harvesting. It must be emphasized, however, that the efficiency gains, yield values and biomass losses reported here were quantified under the specific agro-meteorological conditions of the 2024 growing season (+0.8 °C thermal anomaly and −14% precipitation deficit relative to the 1994–2023 long-term average; Table 13); their absolute magnitude is therefore expected to vary with year-to-year climatic variability and should be re-assessed in subsequent seasons before being adopted as design parameters for commercial-scale mechanization.

Overall, the findings of this study highlight a clear relationship between harvesting technique and the chemical profile of *S. fruticosa*, demonstrating that agricultural practices can influence the balance between essential oil composition and phenolic antioxidant systems. These results provide valuable scientific insight for improving mechanization strategies while maintaining phytochemical quality in medicinal and aromatic plant production systems. It should be emphasized, however, that the present work is based on a single growing season (2024) in one representative Mediterranean-type environment and on three biological replicates per treatment. Therefore, the findings should be regarded as preliminary results that require confirmation under multi-year, multi-location field trials, in which the year × harvesting-method interaction and seasonal climatic variability can be fully incorporated into the statistical model. Future studies should also integrate molecular-level measurements (enzyme activities and terpene-biosynthesis gene expression) to move from the phenotypic, data-driven observations reported here toward a mechanistic understanding of how mechanical harvesting shapes the essential oil and antioxidant profile of *S. fruticosa*.

## Figures and Tables

**Table 1:** Essential oil content (%) and yield (L ha^−1^) of manually and machine-harvested *S. fruticosa*.

Harvesting Methods	Essential Oil Content (%)	Essential Oil Yield (L ha^−1^)
Manual Harvesting	2.03 ± 0.15 ^a^	49.5 ± 0.37 ^a^
Machine Harvesting	1.57 ± 0.22 ^b^	33.3 ± 0.56 ^b^

Note: Mean ± SD (*n* = 3), EO content: *p* < 0.01; EO yield: *p* < 0.001. Different letters (a, b) indicate statistically significant differences between harvesting methods (Tukey’s HSD, *p* < 0.05).

**Table 2:** Essential oil composition of manually and machine-harvested *S. fruticosa*.

Compound	Retention Indices	Manual Harvesting (%)	Machine Harvesting (%)
α-pinene	972	2.77 ± 0.12 ^a^	2.50 ± 0.09 ^b^
α-thujene	976	–	0.45 ± 0.03
Camphene	1027	2.57 ± 0.11 ^a^	2.50 ± 0.08 ^a^
β-pinene	1075	4.03 ± 0.15 ^a^	2.69 ± 0.10 ^b^
Myrcene	1131	2.13 ± 0.08 ^a^	1.35 ± 0.06 ^b^
Limonene	1170	1.64 ± 0.07 ^b^	2.20 ± 0.09 ^a^
1,8-cineole	1188	43.07 ± 0.85 ^a^	21.77 ± 3.48 ^b^
γ-terpinene	1206	0.57 ± 0.04 ^b^	0.85 ± 0.05 ^a^
p-cymene	1218	0.29 ± 0.02 ^b^	1.75 ± 0.07 ^a^
α-terpinolene	1225	0.48 ± 0.03 ^a^	0.43 ± 0.02 ^a^
Thujone	1294	2.83 ± 1.94 ^b^	19.94 ± 0.76 ^a^
cis-sabinol	1391	–	3.94 ± 0.16
Camphor	1490	20.12 ± 2.47 ^a^	16.12 ± 4.73 ^a^
Linalool	1504	0.55 ± 0.04 ^a^	0.33 ± 0.02 ^b^
Linalyl acetate	1514	0.85 ± 0.05 ^a^	0.88 ± 0.04 ^a^
Bornyl acetate	1547	1.68 ± 0.08 ^a^	1.30 ± 0.06 ^b^
Caryophyllene	1565	2.54 ± 0.04 ^b^	5.22 ± 1.47 ^a^
Humulene	1634	1.16 ± 0.04 ^b^	1.36 ± 0.07 ^a^
(+)-4-Carene	1656	1.25 ± 0.07 ^a^	0.29 ± 0.02 ^b^
Endo-borneol	1664	0.99 ± 0.05 ^a^	0.96 ± 0.04 ^a^
Geranyl acetate	1704	0.14 ± 0.01 ^a^	0.05 ± 0.01 ^b^
Geraniol	1785	0.11 ± 0.01 ^a^	0.06 ± 0.01 ^b^
Caryophyllene oxide	1914	0.40 ± 0.03 ^b^	1.50 ± 0.06 ^a^
Viridiflorol	1987	0.85 ± 0.08 ^b^	5.84 ± 0.44 ^a^
Thymol	2036	0.03 ± 0.02 ^b^	0.13 ± 0.01 ^a^
Eugenol	2042	0.02 ± 0.01 ^b^	0.04 ± 0.01 ^a^
**Total**		**95.73**	**94.57**

Note: Mean ± SD (*n* = 3). Different superscript letters (a, b) within the same row indicate statistically significant differences between harvesting methods (Tukey’s HSD, *p* < 0.05). “–” = not detected. Welch *t*-test: 6 compounds *p* < 0.001, 9 at *p* < 0.01, 2 at *p* < 0.05, 7 n.s. (Table 12).

**Table 3:** Major components of the essential oils of manually and machine-harvested *S. fruticosa*.

Compound	Manual Harvesting (%)	Machine Harvesting (%)
1,8-cineole	43.07 ± 0.85 ^a^	21.77 ± 3.48 ^b^
Camphor	20.12 ± 2.47 ^a^	16.12 ± 4.73 ^a^
Thujone	2.83 ± 1.94 ^b^	19.94 ± 0.76 ^a^
Caryophyllene	2.54 ± 0.04 ^b^	5.22 ± 1.47 ^a^
Viridiflorol	0.85 ± 0.08 ^b^	5.84 ± 0.44 ^a^

Note: Mean ± SD (*n* = 3). Different superscript letters (a, b) within the same row indicate statistically significant differences between harvesting methods (Tukey’s HSD, *p* < 0.05).

**Table 4:** Effect of harvesting methods on the chemical class distribution of *S. fruticosa* essential oil.

Chemical Group	Manual Harvesting (%)	Machine Harvesting (%)
Monoterpene hydrocarbons	15.73	16.01
Oxygenated monoterpenes	75.06	69.56
Sesquiterpene hydrocarbons	3.70	6.70
Oxygenated sesquiterpenes	1.25	7.34

**Table 5:** Macro elements of manually and machine-harvested *S. fruticosa*.

Harvesting Methods	Macro Elements				
	P (ppm)	K (ppm)	Ca (ppm)	Mg (ppm)	S (ppm)
Manual Harvesting	1683.13 ± 2.45 ^a^	11,350.17 ± 5.73 ^a^	32,564.92 ± 57.72 ^a^	4960.92 ± 4.26 ^b^	2838.67 ± 11.80 ^a^
Machine Harvesting	1560.66 ± 1.33 ^b^	10,640.23 ± 28.93 ^b^	12,573.18 ± 32.27 ^b^	5894.43 ± 11.11 ^a^	1845.32 ± 2.27 ^b^

Note: Mean ± SD (*n* = 3), P, K, Ca, Mg, S: *p* < 0.001. Different letters (a, b) indicate statistically significant differences between harvesting methods (Tukey’s HSD, *p* < 0.05).

**Table 6:** Micro elements of manually and machine-harvested *S. fruticosa*.

Harvesting Methods	Micro Elements				
	Fe (ppm)	Zn (ppm)	Cu (ppm)	Mn (ppm)	B (ppm)
Manual Harvesting	840.28 ± 2.06 ^a^	15.12 ± 0.54 ^a^	6.69 ± 0.11 ^a^	30.98 ± 0.10 ^b^	40.58 ± 0.09 ^a^
Machine Harvesting	805.52 ± 3.02 ^b^	9.53 ± 0.09 ^b^	4.72 ± 0.09 ^b^	31.71 ± 0.36 ^a^	33.20 ± 0.63 ^b^

Note: Mean ± SD (*n* = 3), Fe, Zn, Cu, B: *p* < 0.001; Mn: *p* < 0.05. Different letters (a, b) indicate statistically significant differences between harvesting methods (Tukey’s HSD, *p* < 0.05).

**Table 7:** Antioxidant Activity of manually and machine-harvested *S. fruticosa*.

Harvesting Methods	Antioxidant Activity (mg TE mL^−1^)
Manual Harvesting	0.093 ± 0.001 ^a^
Machine Harvesting	0.096 ± 0.001 ^a^

Note: Mean ± SD (*n* = 3), not significant (*p* > 0.05). Means sharing the same superscript letter (a) are not significantly different between harvesting methods (Tukey’s HSD, *p* > 0.05).

**Table 8:** Ferric-Reducing Antioxidant Power (FRAP) of manually and machine-harvested *S. fruticosa*.

Harvesting Methods	FRAP (µmol Fe^2+^ g^−1^ Extract)
Manual Harvesting	382 ± 2.45 ^a^
Machine Harvesting	336 ± 3.08 ^a^

Note: Mean ± SD (*n* = 3), not significant (*p* > 0.05). Means sharing the same superscript letter (a) are not significantly different between harvesting methods (Tukey’s HSD, *p* > 0.05).

**Table 9:** Total Phenolic Content (TPC) of manually and machine-harvested *S. fruticosa*.

Harvesting Methods	TPC (mg GAE g^−1^ Extract)
Manual Harvesting	134.6 ± 2.12 ^a^
Machine Harvesting	129.3 ± 1.85 ^a^

Note: Mean ± SD (*n* = 3), not significant (*p* > 0.05). Means sharing the same superscript letter (a) are not significantly different between harvesting methods (Tukey’s HSD, *p* > 0.05).

**Table 10:** Total Flavonoid Content (TFC) of manually and machine-harvested *S. fruticosa*.

Harvesting Methods	TFC (mg QE g^−1^ Extract)
Manual Harvesting	22.7 ± 1.34 ^a^
Machine Harvesting	25.2 ± 1.48 ^a^

Note: Mean ± SD (*n* = 3), not significant (*p* > 0.05). Means sharing the same superscript letter (a) are not significantly different between harvesting methods (Tukey’s HSD, *p* > 0.05).

**Table 13:** Monthly mean air temperature (T, °C), total precipitation (P, mm) and mean relative humidity (RH, %) during the 2024 growing season at the Goztepe-Karaman site compared with the 1994–2023 long-term average (Turkish State Meteorological Service, station 17246).

Month	T (°C)		P (mm)		RH (%)	
	2024	LT avg.	2024	LT avg.	2024	LT avg.
March	7.1	6.4	28.4	36.2	58	61
April	12.4	11.6	24.8	41.5	52	57
May	17.5	16.3	26.1	44.7	49	54
June	22.1	20.8	18.3	22.6	45	48
July	26.0	24.7	4.1	6.8	38	42
May–June	17.0	16.0	101.7	151.8	48	52

## Data Availability

The data presented in this study are available in the article.

## Abbreviations

The following abbreviations are used in this manuscript:

EO	Essential Oil
MAP	Medicinal and Aromatic Plant
GC–MS	Gas Chromatography–Mass Spectrometry
ICP–MS	Inductively Coupled Plasma–Mass Spectrometry
DPPH	2,2-Diphenyl-1-picrylhydrazyl
FRAP	Ferric Reducing Antioxidant Power
TPC	Total Phenolic Content
TFC	Total Flavonoid Content
TE	Trolox Equivalent
GAE	Gallic Acid Equivalents
QE	Quercetin Equivalents
LSD	Least Significant Difference
HSD	Honestly Significant Difference
ha	Hectare
mM	Millimolar
